# Supplementary material for: Telemedicine-Based Specialized Care Improves the Outcome of Anticoagulated Individuals with Venous Thromboembolism—Results from the thrombEVAL Study
Source: J Clin Med. 2020 Oct 13;9(10):3281. doi: 10.3390/jcm9103281 (PMC7602093; doi:10.3390/jcm9103281)
Supplement: Supplementary file 1 [file jcm-09-03281-s001.pdf]

## SUPPLEMENTAL APPENDIX

# Telemedicine-based specialized care improves the outcome of anticoagulated individuals with venous thromboembolism – Results from the thrombEVAL study

Running title: Telemedicine-based specialized care in VTE

Karsten Keller[1,2], Sebastian Göbel[2,3], Vincent ten Cate[4,1], Marina Panova-Noeva[1,3], Lisa Eggebrecht[4,1], Markus Nagler[1,4], Meike Coldewey[2,1], Maike Foebel[1], Christoph Bickel[5], Michael Lauterbach[6], Christine Espinola-Klein[2], Karl J. Lackner[8,3], Hugo ten Cate[9,1], Thomas Münzel[2,3,1], Philipp S. Wild[4,1,3], and Jürgen H. Prochaska[4,1,3]

[1] Center for Thrombosis and Hemostasis (CTH), University Medical Center of the Johannes Gutenberg-University Mainz, Mainz, Germany

[2] Center of Cardiology, Cardiology I, University Medical Center of the Johannes Gutenberg-University Mainz, Mainz, Germany

[3] German Center for Cardiovascular Research (DZHK), Partner Site Rhine Main, Mainz, Germany

[4] Preventive Cardiology and Preventive Medicine – Center of Cardiology, University Medical Center of the Johannes Gutenberg-University Mainz, Mainz, Germany

[5] Department of Medicine I, Federal Armed Forces Central Hospital Koblenz, Koblenz, Germany

[6] Department of Medicine 3, Barmherzige Brüder Hospital, Trier, Germany

[7] Center for General Medicine and Geriatric Medicine, University Medical Center of the Johannes Gutenberg-University Mainz, Mainz, Germany

[8] Institute of Clinical Chemistry and Laboratory Medicine, University Medical Center of the Johannes Gutenberg-University Mainz, Mainz, Germany

[9] Thrombosis Center Maastricht, Cardiovascular Research Institute Maastricht and Maastricht University Medical Center, Maastricht, the Netherlands

**Supplemental Methods**

In the RMC cohort, patients' OAC-treatment-management was continued by the physicians in charge (i.e. general practitioner, but also specialists) after study enrolment. Regarding the observational character of the thrombEVAL study, decisions were left to the discretion of the treating physicians in charge and were not influenced by the study protocol or the investigators.[1] In the RMC in Germany, the physicians take the blood samples ambulatory during standard visits at the doctor's office. Blood samples were analysed in the laboratory cooperating with the respective physician. Afterwards, depending the laboratory results, the dosing was performed by the physician contacting the patient.

In contrast, the coagulation service was operated by the Center of Thrombosis and Hemostasis, University Medical Center Mainz (Germany) (**Figure S1 in the supplementary appendix**). In the CS, specialized staff consisting of physicians and nurses, who were trained in hemostasis, shouldered the responsibility for patients' OAC-treatment adjustments. Standardized visits for anticoagulation control at 16 urban and rural service points at fixed consultation hours were carried out. Additionally, the CS staff performed home visits for frail and disabled patients, depending on their physical status and the individual need.[1,2] The OAC treatment-relevant information, which included standardized assessment of clinical status at each visit, identification of complications and the level of international normalized ratio (INR) was assessed and recorded digitally in a web-based electronic patient file (Portavita B.V., Amsterdam, the Netherlands).[1] The blood samples were analyzed in a central reference laboratory at the university medical center Mainz (Germany), which facilitated comparability of INR values. The staff performed individual VKA dosing by incorporation of all relevant information in the electronic file, with support of computer-assisted dosing algorithms and subsequent professional optimization, if necessary.[1] Additionally, the electronic file provided the interface for patient management in collaboration with general practitioners, specialists and health care institutions.[1,2] Additionally, patients with deviated INR-values from the INR target range were contacted by telephone and precise management recommendations were given by the CS. Telemedicine CS was based on

the experiences of other INR-clinics. The RMC as well as the telemedicine-based CS are described in detail in the design paper of the thrombEVAL study programme [2].

In the future, demographic changes will increase the proportion of older patients. Risk of VTE increases exponentially with aging[3-7] and an incline of individuals with VTE events with the need for OAC treatment has to be expected. Additionally, with an aging population numbers of multi-morbid patients is growing.[3-9] Future concepts have to take these changes into account, particularly changes in OAC treatment regarding aging process, therapy adherence, mental and physical capabilities and influence of co-morbidities on treatment.[10,11] Therefore, VKA-treatment will maintain its position in OAC treatment.

**Supplemental Results**

**Supplemental Table S1: Inclusion and exclusion criteria of the thrombEVAL study.**

| **Inclusion criteria** | | **Exclusion crieria** |
|---|---|---|
| **Regular medical care** | **Coagulation service** | |
| Age ≥18 years | | Age <18 years |
| Written informed consent | | Withdrawal of priorly given consent |
| Performance of VKA therapy ≥4 months | | Contraindications to VKA treatment (e.g. pregnancy or known hypersensitivity) |
| | Indication for at least 3 months duration of VKA treatment | Participation in other clinical trials |

Abbreviations: VKA indicates vitamin K antagonist treatment

**Supplemental Table S2. Baseline characteristics of VTE patients in regular medical care (RMC) and coagulation service (CS) groups after propensity score weighting.**

| Variable | Coagulation Service | Regular medical care | P-value |
|---|---|---|---|
| Age (years; median IQR) | 73.0 (60.5/81.0) | 72.0 (60.0/79.0) | 0.32 |
| Sex (Men) | 51.6% (193/375) | 53.9% (194/360) | 0.57 |
| | | | |
| **Classical cardiovascular risk factors** | | | |
| Arterial hypertension | 73.1% (274/375) | 71.9% (259/360) | 0.76 |
| Diabetes | 25.5% (96/375) | 26.7% (96/360) | 0.74 |
| Dyslipidemia | 46.5% (174/375) | 51.4% (185/360) | 0.23 |
| Family history of myocardial infarction | 38.0% (142/375) | 39.7% (143/360) | 0.66 |
| | | | |
| **Concomitant diseases** | | | |
| Arterial fibrillation | 49.8% (186/375) | 46.1% (166/360) | 0.37 |
| Chronic lung disease | 29.5% (111/375) | 28.3% (102/360) | 0.75 |
| Coronary artery disease | 33.2% (124/375) | 35.3% (127/360) | 0.58 |
| Heart failure | 38.4% (144/375) | 35.3% (127/360) | 0.42 |
| Peripheral artery disease | 29.0% (109/375) | 21.9% (79/360) | 0.044 |
| Renal disease | 24.5% (92/375) | 23.6% (85/360) | 0.80 |
| | | | |
| **OAC therapy management** | | | |
| Self measurement and management | 17.4% (65/375) | 15.8% (57/360) | 0.60 |
| | | | |
| **Other Medication** | | | |

| | | | |
|---|---|---|---|
| Anti-platelet agents | 13.1% (49/375) | 18.1% (65/360) | 0.088 |
| Proton pump inhibitor | 34.2% (128/375) | 35.0% (126/360) | 0.84 |
| Amiodarone | 4.8% (18/375) | 5.8% (21/360) | 0.56 |

Data are expressed as relative and absolute frequencies in binary variables. Variables are shown as median with $25^{th}/75^{th}$ percentiles. Double entries were possible for those study participants in CS cohort with prior treatment duration in RMC.

Abbreviations: IQR indicates inter-quartile range.

**Table S3. Comparison of outcome in regular medical care and coagulation service by adjusted effect measures in a competing risks analysis.**

| Variables of adjustment | Crude model | | Adjustment for age and sex | | Adjustment for age, sex and Charlson-Index | | Adjustment for age, sex, diabetes, arterial hypertension, dyslipidemia, smoking, family history of myocardial infarction, heart failure, peripheral artery disease, history of stroke/TIA, atrial fibrillation, chronic lung disease, renal disease, home visits for treatment, anticoagulant treatment self-management, HAS-BLED-Score, $CHA_2DS_2$-VASc-Score | |
|---|---|---|---|---|---|---|---|---|
| | **Hazard Ratio (95% CI)** | **P Value** | **Hazard Ratio (95% CI)** | **P Value** | **Hazard Ratio (95% CI)** | **P Value** | **Hazard Ratio (95% CI)** | **P Value** |
| Primary outcome | 5.85 (2.33-14.71) | <0.001 | 5.44 (2.16-13.74) | <0.001 | 5.96 (2.28-15.62) | 0.00028 | 4.96 (1.50-16.34) | 0.0086 |
| Clinically relevant bleeding | 5.43 (1.92-15.34) | 0.0014 | 5.09 (1.79-14.46) | 0.0022 | 6.13 (2.06-18.20) | 0.0011 | 5.13 (1.30-20.26) | 0.020 |
| Thromboembolic events | 6.51 (1.54-27.50) | 0.011 | 6.12 (1.42-26.42) | 0.015 | 6.16 (1.38-27.46) | 0.017 | 4.27 (0.73-24.97) | 0.11 |
| Hospitalization | 1.84 (1.34-2.53) | <0.001 | 1.76 (1.28-2.44) | <0.001 | 1.83 (1.28-2.61) | 0.00090 | 1.82 (1.18-2.82) | 0.0066 |

Hazard ratios for the endpoints were presented with different adjustments. Hazard ratios of RMC are provided as multiples in comparison to the reference group of CS. Bleeding events were categorized regarding to RELY criteria. Significant differences were seen if p was <0.05.

Abbreviations: CI indicates confidence interval

**Supplemental Figure S1: Work flow of the telemedicine-based coagulation service.**

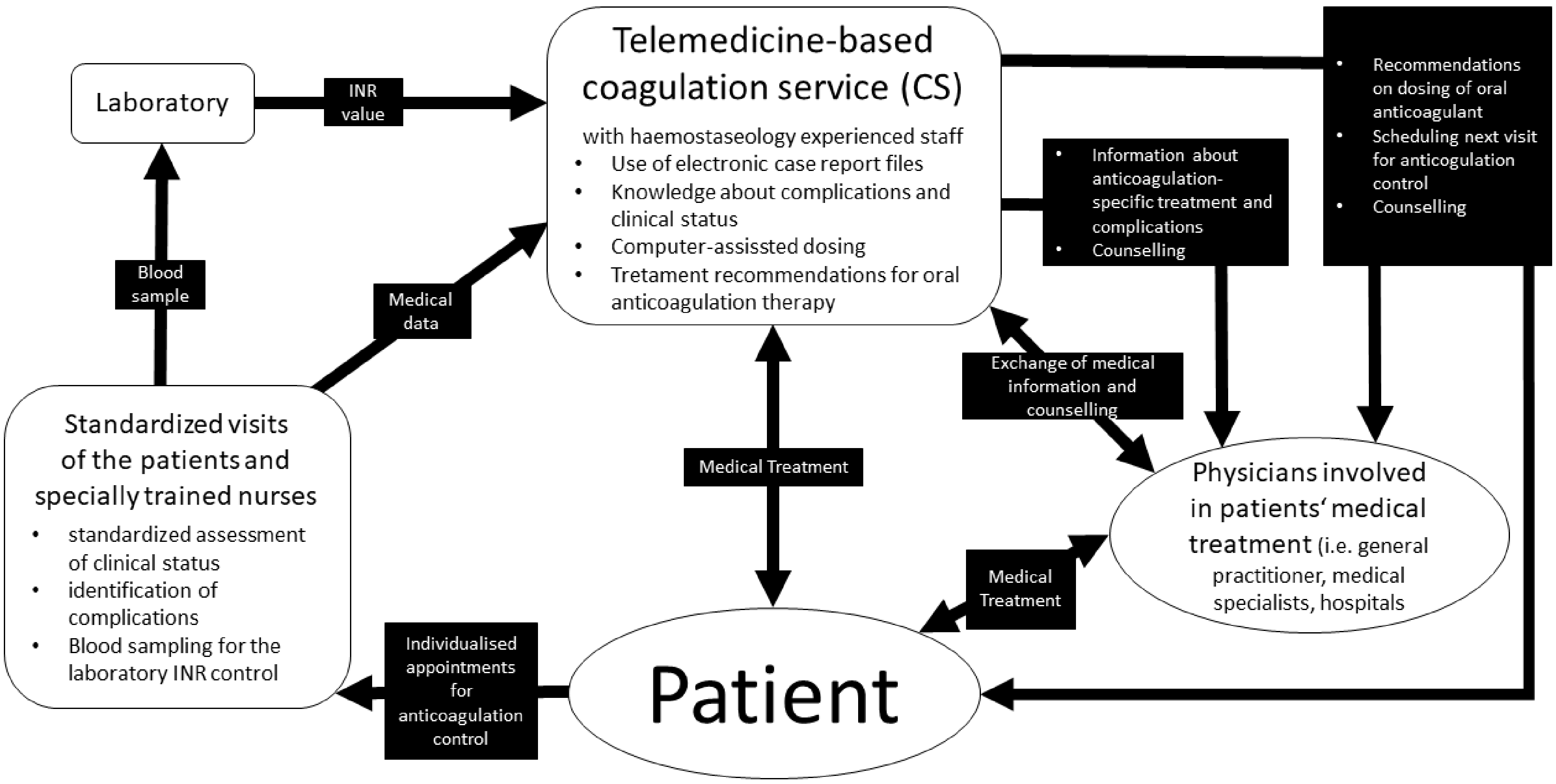

**Supplemental Figure S2.**

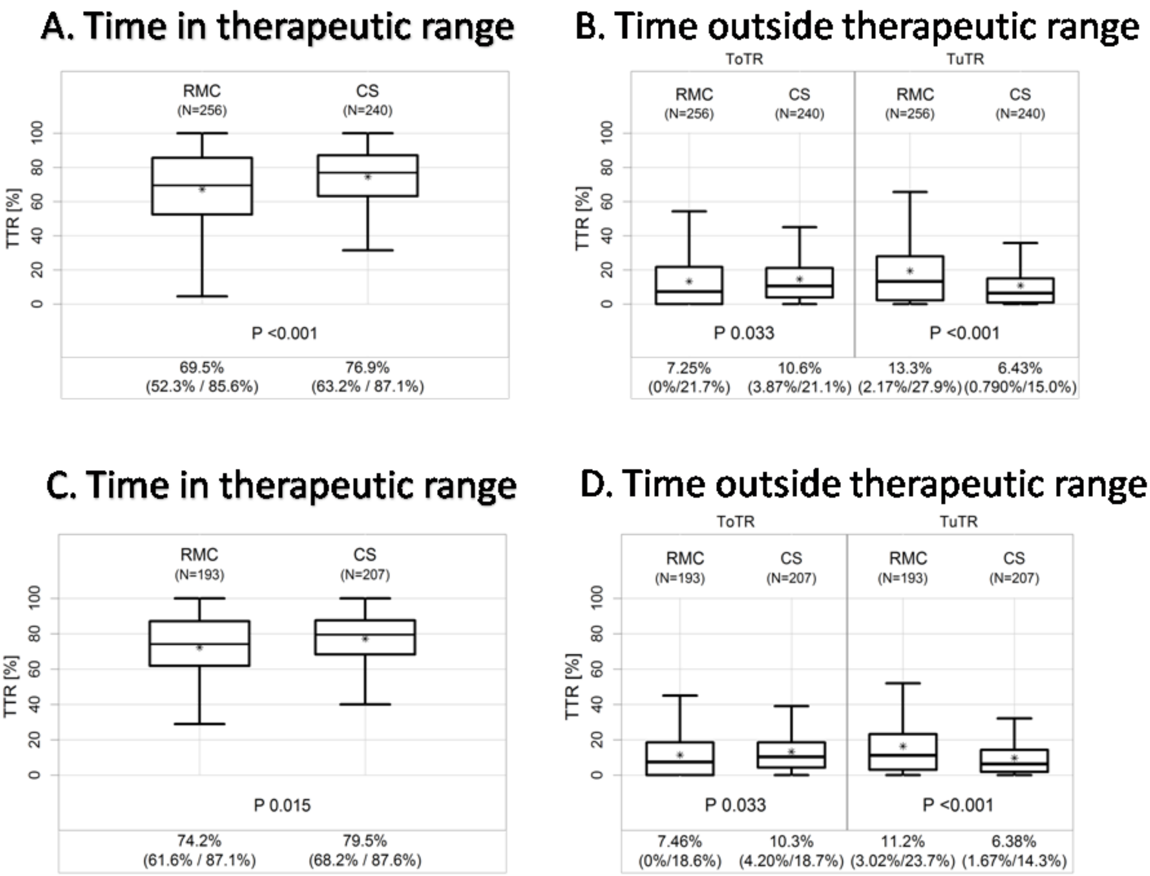


**A: Overall patients' quality of oral anticoagulation therapy in regular medical care (RMC) and coagulation service (CS): Time in therapeutic range (TTR).** TTR was calculated according to linear interpolation method and presented as median (first quartile/third quartile); *P* value for z-test. Mean TTR values are depicted graphically as asterisks within box-plots. *TTR variability is expressed by median absolute deviation (MAD), *P* value for Ansari-Bradley-test.

**B: Overall patients' quality of oral anticoagulation therapy in regular medical care (RMC) and coagulation service (CS): Time outside therapeutic range.** Percentage of Time outside therapeutic range is calculated according to linear interpolation method and presented as median (first quartile/third quartile); *P* value for z-test. ToTR means Time over therapeutic range,TuTR has the meaning Time under therapeutic range.

**C: Stable adjusted patients' quality of oral anticoagulation therapy in regular medical care (RMC) and coagulation service (CS): Time in therapeutic range (TTR).** TTR was calculated

according to linear interpolation method and presented as median (first quartile/third quartile); *P* value for z-test. Mean TTR values are depicted graphically as asterisks within box-plots. *TTR variability is expressed by median absolute deviation (MAD), *P* value for Ansari-Bradley-test.

**D: Stable adjusted patients' quality of oral anticoagulation therapy in regular medical care (RMC) and coagulation service (CS): Time outside therapeutic range.** Percentage of Time outside therapeutic range is calculated according to linear interpolation method and presented as median (first quartile/third quartile); *P* value for z-test. ToTR means Time over therapeutic range,TuTR has the meaning Time under therapeutic range.

**Supplemental Figure S3. Comparison of clinical endpoints in individuals with VTE by health care model.**

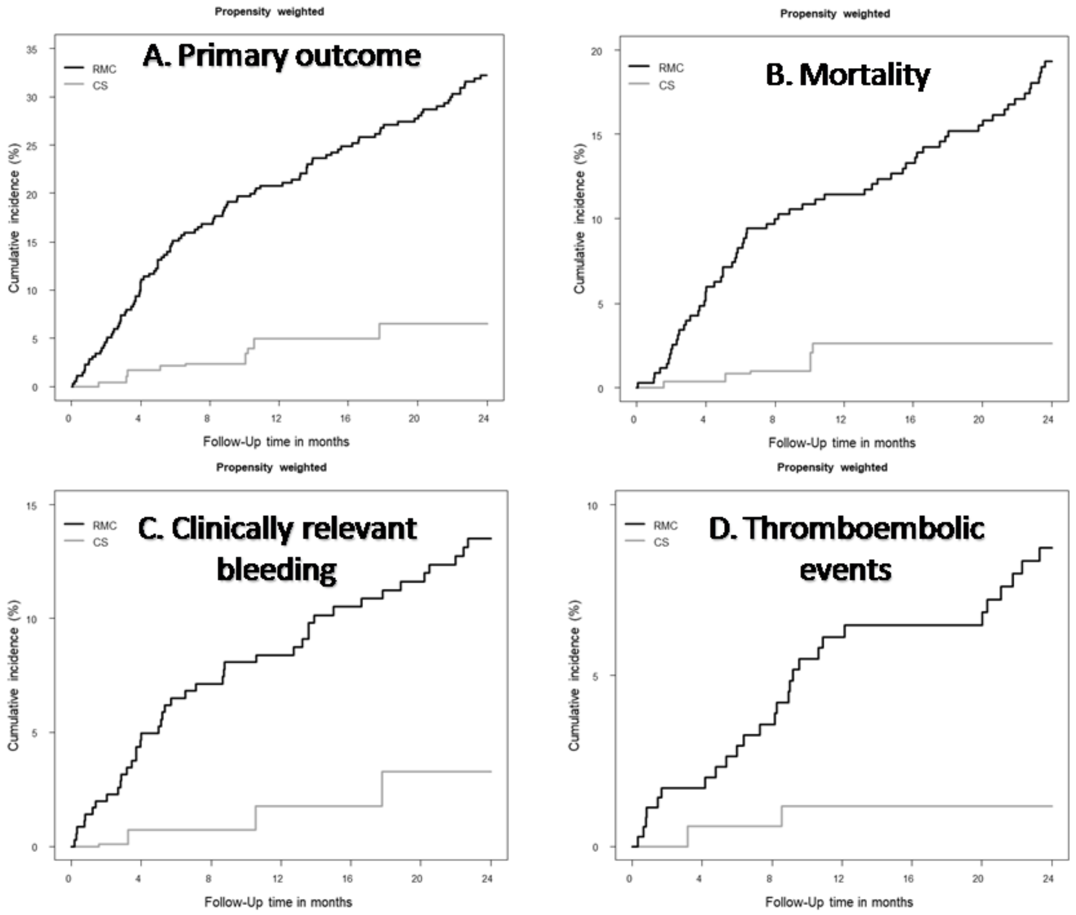


Crude cumulative incidence plots of adverse events in regular medical care (RMC) and coagulation service (CS): RMC in black and CS in grey colour for the adverse events under OAC treatment during 2 years follow-up period.

**Supplemental Figure S4: Primary outcome in coagulation service versus regular medical care according to subgroups.**

| Factor | Group | HR | 95% CI | p | p for interaction |
|---|---|---|---|---|---|
| Sex | Women | 8.64 | [3.11,23.96] | <0.001 | 0.26 |
| | Men | 4.17 | [1.79, 9.74] | <0.001 | |
| Obesity | No | 5.94 | [2.73,12.92] | <0.001 | 0.89 |
| | Yes | 5.58 | [1.70,18.33] | 0.0046 | |
| CAD | No | 7.99 | [3.44,18.56] | <0.001 | 0.11 |
| | Yes | 2.59 | [0.92, 7.26] | 0.071 | |
| Heart failure | No | 5.01 | [2.26,11.10] | <0.001 | 0.93 |
| | Yes | 4.9 | [1.53,15.67] | 0.0075 | |
| Hypertension | No | 10.76 | [2.55,45.44] | 0.0012 | 0.30 |
| | Yes | 4.54 | [2.19, 9.43] | <0.001 | |
| Prior PE | No | 4.85 | [2.09,11.28] | <0.001 | 0.57 |
| | Yes | 6.98 | [2.50,19.50] | <0.001 | |
| Renal disease | No | 8.26 | [3.32,20.53] | <0.001 | 0.15 |
| | Yes | 3.04 | [1.19, 7.77] | 0.020 | |
| Any prior bleeding | No | 5.74 | [2.74,12.03] | <0.001 | 0.83 |
| | Yes | 5.24 | [1.27,21.65] | 0.022 | |
| TTR > 70 | No | 5.03 | [1.79,14.14] | 0.0022 | 0.95 |
| | Yes | 5.9 | [2.46,14.14] | <0.001 | |
| ASS | No | 6.68 | [3.08,14.46] | <0.001 | 0.44 |
| | Yes | 4.05 | [1.20,13.69] | 0.025 | |
| PPI | No | 6.39 | [2.56,15.96] | <0.001 | 0.86 |
| | Yes | 5.52 | [2.19,13.95] | <0.001 | |
| **Whole Sample** | | **5.87** | **[3.06,11.26]** | **<0.001** | |

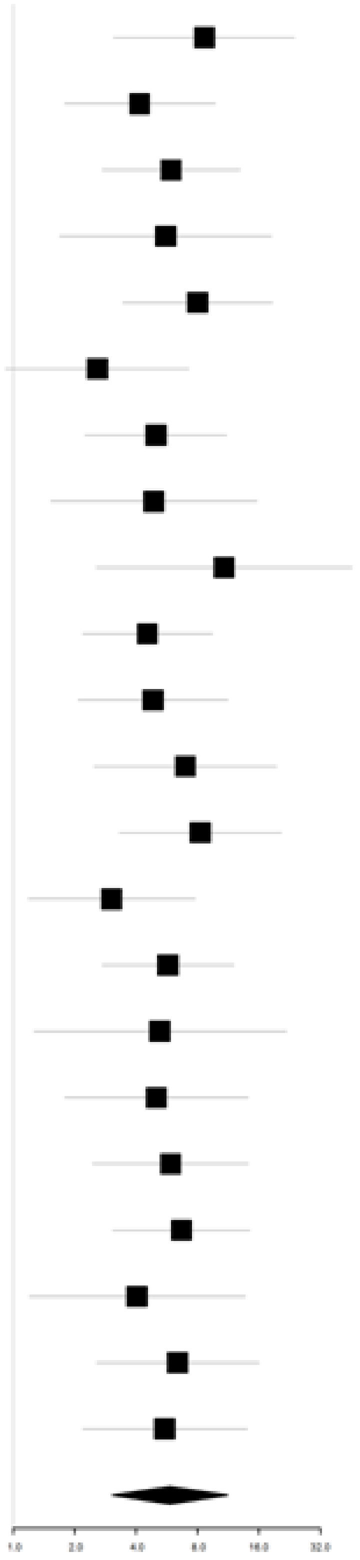

**Supplemental Text. List of Study Investigators** (representatives of the study centers in alphabetic order).

Bickel C, MD, Kowitz C, Schmidt-Borko KM (Koblenz; Germany); Elsner M, MD, Hampel C, MD (Bad Kreuznach, Germany); Brako M, MD, Nietsch M, MD (Bad Kreuznach, Germany); Münchbach M, MD, Schwacke H, MD (Speyer, Germany); Meier-Willersen H, MD, Weis-Reinecke C, MD (Speyer, Germany); Heckmann J, MD (Bad Münster am Stein, Germany); Keck M, MD (Bad Münster am Stein, Germany); Schmidt W, MD (Bingen, Germany); Efferenn C, MD, Kratz T, MD (Simmern, Germany); Hardt R, MD, Wiechelt J, MD (Mainz, Germany), Jung, M, MD, Remplewski K, MD (Mainz, Germany), Dippold W, MD, Mertens D, MD (Mainz, Germany); Coldewey M, MD, Göbel S,MD, Hendelmeier M, MD, Keller K, MD, Münzel T, MD, Prochaska J, MD, Wild PS, MD, MSc (Mainz, Germany); Hauptmann, KE, MD; Lauterbach M, MD, Ulrich E, MD (Trier, Germany); Elsner S, MD, Küpper J, MD (Andernach, Germany); Inselmann G, MD (Ludwigshafen, Germany); Raedle J, MD (Kaiserslautern, Germany); Baldzhiev V, MD, Zotz R, MD (Bitburg, Germany), Hügl B, MD (Neuwied, Germany); Stefan V, MD, von Korn, H, MD (Neustadt an der Weinstraße, Germany); Schürmeyer TH, MD (Trier, Germany).

## References


1. Prochaska, J.H.; Gobel, S.; Keller, K.; Coldewey, M.; Ullmann, A.; Lamparter, H.; Schulz, A.; Schinzel, H.; Bickel, C.; Lauterbach, M., et al. e-Health-based management of patients receiving oral anticoagulation therapy: results from the observational thrombEVAL study. *J Thromb Haemost* **2017**, *15*, 1375-1385, doi:10.1111/jth.13727.
2. Prochaska, J.H.; Coldewey, M.; Gobel, S.; Keller, K.; Hendelmeier, M.; Konstantinides, S.; Munzel, T.; Wild, P.S.; thromb, E.S.G. Evaluation of oral anticoagulation therapy: rationale and design of the thrombEVAL study programme. *Eur J Prev Cardiol* **2015**, *22*, 622-628, doi:10.1177/2047487314527852.
3. Kyrle, P.A.; Minar, E.; Bialonczyk, C.; Hirschl, M.; Weltermann, A.; Eichinger, S. The risk of recurrent venous thromboembolism in men and women. *N Engl J Med* **2004**, *350*, 2558-2563, doi:10.1056/NEJMoa032959.
4. Oger, E. Incidence of venous thromboembolism: a community-based study in Western France. EPI-GETBP Study Group. Groupe d'Etude de la Thrombose de Bretagne Occidentale. *Thromb Haemost* **2000**, *83*, 657-660.
5. Silverstein, M.D.; Heit, J.A.; Mohr, D.N.; Petterson, T.M.; O'Fallon, W.M.; Melton, L.J., 3rd. Trends in the incidence of deep vein thrombosis and pulmonary embolism: a 25-year population-based study. *Arch Intern Med* **1998**, *158*, 585-593.

6. Stein, P.D.; Hull, R.D.; Kayali, F.; Ghali, W.A.; Alshab, A.K.; Olson, R.E. Venous thromboembolism according to age: the impact of an aging population. *Arch Intern Med* **2004**, *164*, 2260-2265, doi:10.1001/archinte.164.20.2260.
7. Hippisley-Cox, J.; Coupland, C. Development and validation of risk prediction algorithm (QThrombosis) to estimate future risk of venous thromboembolism: prospective cohort study. *BMJ* **2011**, *343*, d4656, doi:10.1136/bmj.d4656.
8. Pell, J.P.; Alcock, J. Monitoring anticoagulant control in general practice: comparison of management in areas with and without access to hospital anticoagulant. *Br J Gen Pract* **1994**, *44*, 357-358.
9. Poller, L.; Keown, M.; Ibrahim, S.; Lowe, G.; Moia, M.; Turpie, A.G.; Roberts, C.; van den Besselaar, A.M.; van der Meer, F.J.; Tripodi, A., et al. An international multicenter randomized study of computer-assisted oral anticoagulant dosage vs. medical staff dosage. *J Thromb Haemost* **2008**, *6*, 935-943, doi:10.1111/j.1538-7836.2008.02959.x.
10. Rombouts, E.K.; Rosendaal, F.R.; Van Der Meer, F.J. Daily vitamin K supplementation improves anticoagulant stability. *J Thromb Haemost* **2007**, *5*, 2043-2048, doi:10.1111/j.1538-7836.2007.02715.x.
11. Prochaska, J.H.; Gobel, S.; Keller, K.; Coldewey, M.; Ullmann, A.; Lamparter, H.; Junger, C.; Al-Bayati, Z.; Baer, C.; Walter, U., et al. Quality of oral anticoagulation with phenprocoumon in regular medical care and its potential for improvement in a telemedicine-based coagulation service inverted question mark results from the prospective, multi-center, observational cohort study thrombEVAL. *BMC Med* **2015**, *13*, 14, doi:10.1186/s12916-015-0268-9.
